# Are antiangiogenics a good ‘partner’ for immunotherapy in ovarian cancer?

**DOI:** 10.1007/s10456-020-09734-w

**Published:** 2020-07-20

**Authors:** Elena García-Martínez, Andres Redondo, Josep Maria Piulats, Analía Rodríguez, Antonio Casado

**Affiliations:** 1 Medical Oncology Department, Hospital Universitario Morales Meseguer, IMIB, Avenida Marques de los Velez, 30008 Murcia, Spain; 2 Medical Oncology Department, Hospital Universitario La Paz-IdiPAZ, Madrid, Spain; 3 Institut Català d’OncologiaMedical Oncology Unit – IDIBELL/OncoBell – CIBERONC, Barcelona, Spain; 4 Roche Farma S.A., Madrid, Spain; 5 Department of Medical Oncology, Hospital Clínico San Carlos, Madrid, Spain

**Keywords:** Ovarian cancer, Angiogenesis, Immune evasion, Immunomodulator, Bevacizumab

## Abstract

Ovarian cancer (OC) is associated with poor survival because there are a limited number of effective therapies. Two processes key to OC progression, angiogenesis and immune evasion, act synergistically to promote tumor progression. Tumor-associated angiogenesis promotes immune evasion, and tumor-related immune responses in the peritoneal cavity and tumor microenvironment (TME) affect neovascular formation. Therefore, suppressing the angiogenic pathways could facilitate the arrival of immune effector cells and reduce the presence of myeloid cells involved in immune suppression. To date, clinical studies have shown significant benefits with antiangiogenic therapy as first-line therapy in OC, as well as in recurrent disease, and the vascular endothelial growth factor (VEGF) inhibitor bevacizumab is now an established therapy. Clinical data with immunomodulators in OC are more limited, but suggest that they could benefit some patients with recurrent disease. The preliminary results of two phase III trials have shown that the addition of immunomodulators to chemotherapy does not improve progression-free survival. For this reason, it could be interesting to look for synergistic effects between immunomodulators and other active drugs in OC. Since bevacizumab is approved for use in OC, and is tolerable when used in combination with immunotherapy in other indications, a number of clinical studies are underway to investigate the use of bevacizumab in combination with immunotherapeutic agents in OC. This strategy seeks to normalize the TME via the anti-VEGF actions of bevacizumab, while simultaneously stimulating the immune response via the immunotherapy. Results of these studies are awaited with interest.

## Introduction

According to 2018 estimates, approximately 300,000 women around the world are diagnosed with ovarian cancer (OC) each year and approximately 185,000 women die from OC [1, 2]. Despite the fact that most patients respond to initial chemotherapy and a few can obtain cure with surgery and first-line chemotherapy, the vast majority of patients with advanced disease will relapse within 2 years after diagnosis. The standard first-line chemotherapy includes at least a platinum and taxane combination [3], usually carboplatin and paclitaxel.

Epithelial OC cannot be considered as a single entity as it encompasses several neoplasms, each with specific clinical pathological characteristics [4]. Specific genomic patterns provide an opportunity for targeted therapy, such as inhibitors of poly ADP ribose polymerase (PARP) in patients with *BRCA1/2* mutations [5], mainly high-grade serous carcinoma, or Ras/Raf/MEK/ERK inhibitors in patients with low-grade serous carcinoma or mucinous ovarian carcinoma [4].

The role played by angiogenesis in the tumor microenvironment (TME), favoring the growth and spread of the tumor is well established [6]. In addition, important advances have been made in the understanding of the “immunity-cancer” cycle, identifying ways in which the tumor escapes immune surveillance, thereby allowing the tumor to grow and metastasize [7]. The “immunity-cancer” relationship is determined by a complex interplay of the intrinsic properties of the tumor (including genetics), host genetics, and environmental factors [7]. OC TME is highly immunosuppressive shielding the tumor from the body’s protective immune cells, and allowing unsuppressed progression [8].

Advances in our understanding of cancer biology have led to the development of angiogenesis inhibitors and immunotherapies as cancer treatments. While the angiogenesis inhibitor bevacizumab is now a common systemic therapy for OC, no immunotherapy has yet been approved for this indication. However, there are currently many clinical trials that are evaluating different immune checkpoint inhibitors in OC, some of them with bevacizumab*.*

The aim of this review is to analyze the possible interaction between angiogenesis and immunomodulation mechanisms specifically in OC and describe the rationale for combining antiangiogenesis agents with immunotherapy based on synergy and complementary mechanisms of action.

## Importance of tumoral angiogenesis in ovarian cancer

Tumor growth and progression rely greatly on the presence of blood vessels to provide oxygen and nutrients and to remove waste products [9]. In physiological conditions angiogenesis is activated by proangiogenic factors, including vascular endothelial growth factor (VEGF), fibroblast growth factor (FGF), transforming growth factor (TGF)-β, epidermal growth factor and angiopoietin, and regulated by the interplay of these proangiogenic factors with antiangiogenic factors [10, 11, 12, 13].

Tumors have an aberrant vasculature that impairs the delivery of oxygen and nutrients to the tumor, resulting in hypoxia and a low intra-tumoral pH [9, 14]. This environment favors selection of more malignant tumor cell clones and the release of more proangiogenic growth factors, generating a vicious cycle of impaired blood vessel formation and poor perfusion [14]. This cycle is mediated in part by the activation of hypoxia-inducible factors (HIFs) that maintain oxygen homoeostasis by stimulating vasodilation and stimulating angiogenesis [15]. Additionally, HIF signaling improves the metabolic fitness of tumor cells so that they preferentially take up nutrients, starving healthy stromal cells in their vicinity [16].

VEGF is the most studied angiogenesis mediator [6], and its receptor is expressed frequently on OC cells [17]. VEGF promotes proliferation, survival and migration of endothelial cells (ECs) and is essential for blood vessel formation [14]. Increased VEGF expression in OC is a marker of cancer stage and grade [18], tumor progression [17] and lower survival rates [4].

Ascites is common in women with advanced OC [19], and the ascites fluid is rich in proangiogenic factors including VEGF and immunosuppressive cells [20, 21]. The concentration of proangiogenic factors in ascites is a marker of OC tumor invasiveness and a prognostic indicator of worse outcome in OC [20, 22, 23].

## Importance of the immune system in ovarian cancer

OC has a unique relationship with the immune system, which can be partially attributed to its site in the peritoneal cavity, as well as the characteristics of the TME.

The peritoneal environment supports the dissemination of OC cells, via the omental vasculature and the peritoneal fluid itself. Even in the early stages of OC, when the tumor is confined to the ovary, cancer cells are detectable in peritoneal lavage fluid [24]. Peritoneal fluid is awash with a range of immune-related cells, including T cells and tumor-associated macrophages (TAMs), which contribute to tumor cell proliferation and immune evasion [25].

In addition, ‘milky spots’ on the omentum are clusters of leukocytes similar to follicles of secondary lymphoid tissue that collect antigens and pathogens from the peritoneal cavity and promote an immune response. When OC cells colonize the omentum, the milky spots initially proliferate and grow, primarily driven by macrophage recruitment. However, despite this initial protective response, tumor cells generally grow unchecked in the omentum, presumably because the OC cells are able to suppress the local immune reaction or evade detection [26].

Similar immune evasion processes appear to be at play within the primary ovarian tumor, where the TME modulates tumor cell-induced immunosuppression. TAMs, myeloid-derived suppressor cells (MDSC) and regulatory T cells (Treg) are considered crucial for the maintenance of an immunosuppressive TME [24]. TAMs are similar in structure and function to normal peritoneal macrophages but they show upregulated levels of genes controlling extracellular matrix remodeling. So they might promote cancer progression by adjusting the matrix of the TME and facilitating the invasion of cancer cells into surrounding tissues [25]. These TAMs are M2 polarized, express CD163 surface marker and are activated by IL-13 and IL-4 [27]. Tregs produce immunosuppressive chemokines such as IL-10 and TGF-β, and express membrane-associated receptors involved in immune evasion, such as cytotoxic T-lymphocyte antigen 4 (CTLA-4) [28].

Other tumor-infiltrating lymphocyte (TILs) that may be present in the TME include CD4 + T-helper cells and CD8 + cytotoxic T-lymphocytes. CD8 + cells would normally have antitumor activity, but these effects are suppressed by the upregulation of immune checkpoint molecules. Within the TME, T-cell receptor sensitization and interferon (IFN)-γ secretion make the tumor cell and the antigen-presenting cell express the programmed cell death-1 (PD-1) receptor and its ligand (PD-L1) on their surfaces. Along with increased PD-1 expression, the upregulation of the lymphocyte activation gene 3 (LAG3) negatively affects the function of the CD8 + lymphocytes, greatly diminishing their cytotoxic activity against the tumor [24]. It should be noted that lymphocyte infiltration is histotype-specific [29]. CD8 + TILs were observed in ~ 50% of patients with mucinous or clear cell histologies but were more common in high-grade serous (in ~ 83% of patients), low-grade serous (~ 73%), and endometrioid (~ 72%) histotypes [29].

The expanding knowledge based on the TME in OC has led to research into potential biomarkers of response to different therapies. However, the TME has proven to be heterogeneous between the primary ovarian tumor and individual metastases [30], and this intra- and inter-tumor heterogeneity presents a challenge for interpreting the clinical significance of different biomarkers in biopsy samples [31].

The importance of TILs as a favorable prognostic biomarker in OC seems unquestionable since the publication of seminal research by Zhang and colleagues in 2003 [32]. The prognostic value of CD8 + TILs is independent of residual disease following surgical cytoreduction [29]. These cells have proven their prognostic significance in a range of OC types including high-grade serous, endometrioid, and mucinous subtypes, but not in patients with low-grade serous or clear cell cancer [29].

Data are contradictory regarding the prognostic value of PD-L1 in patients with OC; there are studies relating PD-L1 expression with a favorable prognosis [33, 34, 35] and with a poor prognosis [34, 36]. This discrepancy is probably due to the absence of an agreed system of immunohistochemical assessment, different cut points, different types of antibodies, and differential expression in PD-L1 between cells types, such as stroma cells, epithelium, and macrophages [33, 37].

**Fig. 1:**
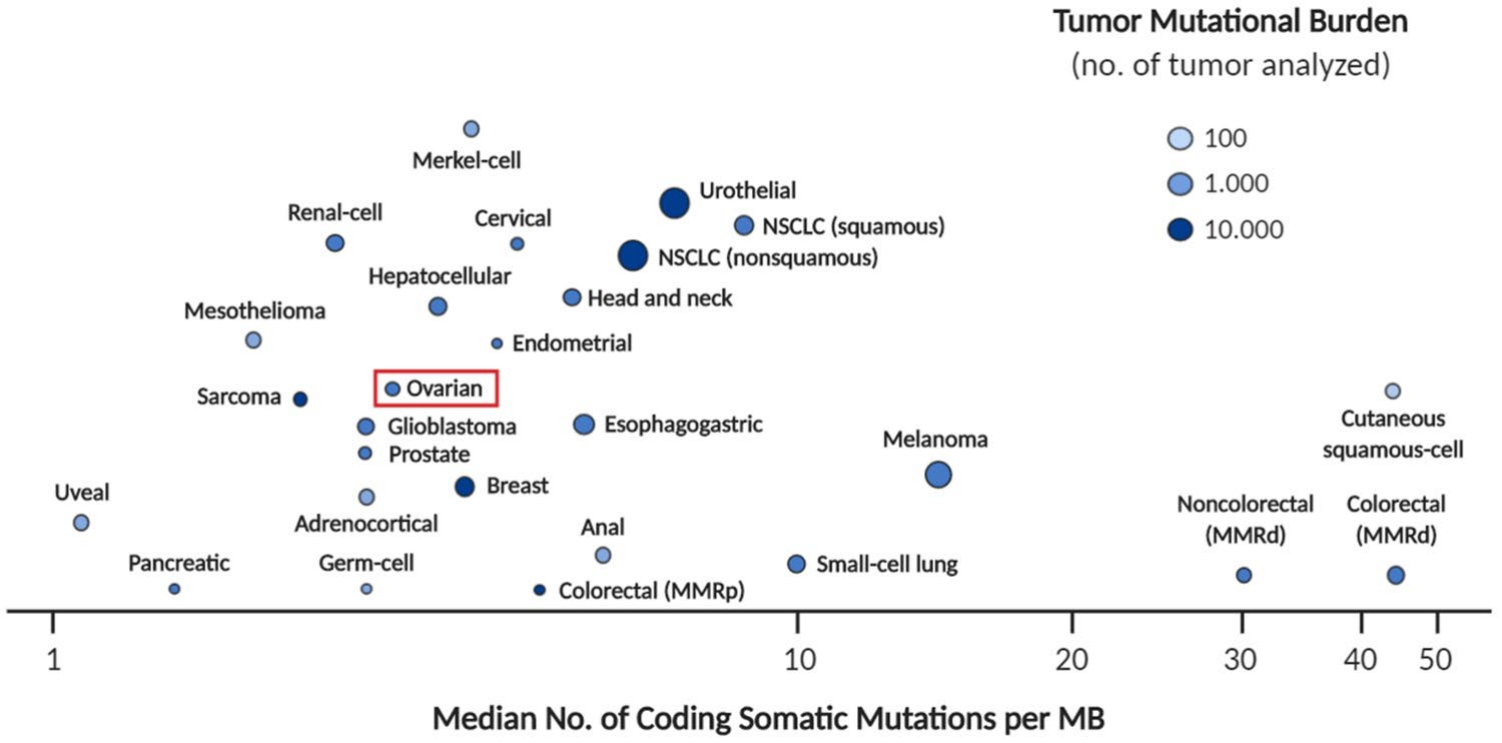
Mutational load in different types of solid tumors [40]. *MMRd* mismatch repair-deficient; *MMRp* mismatch repair-proficient; *no* number; *NSCLC* non-small-cell lung cancer

Mutational load has been used as a biomarker for response to anti–PD-1/PD-L1 treatment in some types of solid tumors [38]. It is most likely that the greater load of tumor-specific neoantigens in the most mutated lesions favors the recruitment of a greater number of TILs [39]. Since the mutational load in OC is lower than in many other types of solid tumor (Fig. 1) [40], the usefulness of this biomarker may be limited in the ovarian setting; however, mutational quality may be of more value since certain mutations confer a greater effect on lymphocyte recruitment than others.

## Biological rationale for the antiangiogenic-immunotherapy combination in cancer

Within the TME, tumor angiogenesis and immune suppression play interconnected roles in promoting tumor progression and metastasis [41].

**Fig. 2:**
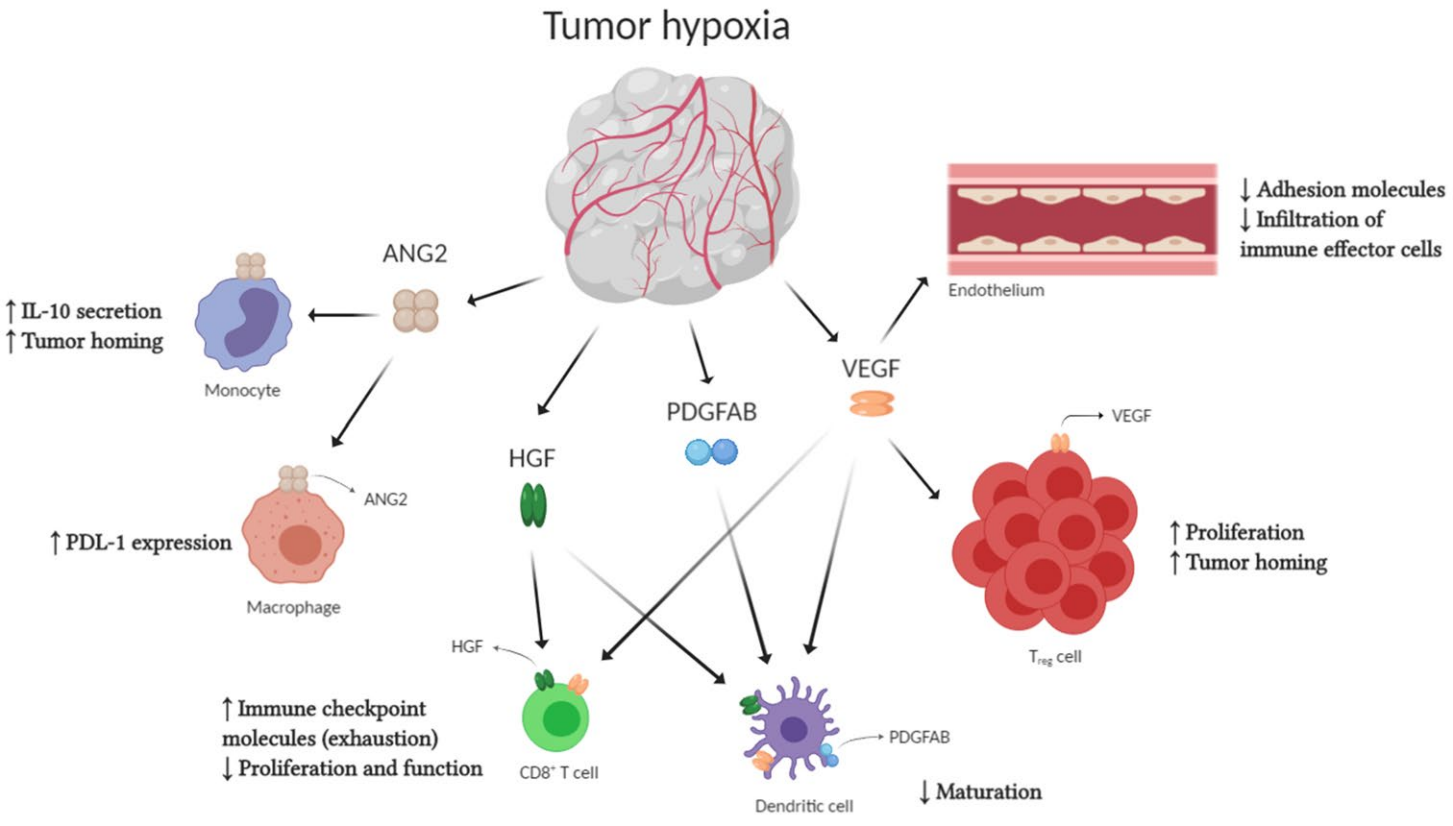
The effect of tumor angiogenic factors on immune cells and the vascular endothelium [42]. Modified from Khan and Kerbel, 2018. *ANG2* angiopoietin 2; *HGF* hepatocyte growth factor; *IL-10* interleukin 10; *PD-L1* programmed death-ligand 1; *PDGFAB* platelet-derived growth factors; *Treg* regulatory T cell; *VEGF* vascular endothelial growth factor

The cytokines and angiogenic factors released by TME cells, such as VEGF, TGFβ and prostaglandin E_2_ (PGE_2_), mediate immunosuppression by reducing antigen presentation to T cells and the effector response of T cells [9]. VEGF in particular modulates immunosuppression by directly and indirectly affecting the function of cells responsible for innate and adaptive immunity (Fig. 2) [9, 42, 43]. The direct mechanisms include an increased recruitment of Treg cells, inflammatory monocytes. and TAMs that are reprogrammed from the M1 subtype of ‘classically activated’ macrophages with anticancer activity to a pro-tumoral M2 phenotype; DC maturation inhibition, which affects the presentation of antigens and the activation of cytotoxic CD8 + cells; and the proliferation of atypical ECs with immunosuppressor phenotype [9, 42, 43].

Other angiogenic molecules are also involved in these processes. For example, angiopoietin 2 (ANG2) induces immunosuppression by binding to macrophages, where it upregulates PD-L1 [42]. Hepatocyte growth factors (HGF) and platelet derivatives (PDGFAB), bind to DCs, thus suppressing their maturation.

VEGF also indirectly affects the inflammatory response by inducing “endothelial anergy,” whereby the endothelium has a dysfunctional response to inflammatory signals [44]. Normally, inflammatory mediators upregulate adhesion molecules, thereby trafficking immune cells from the blood to sites of inflammation. However, in tumors, VEGF and other angiogenic factors downregulate the expression of different adhesion molecules, such as intercellular adhesion molecule 1 (ICAM1) and vascular cell adhesion molecule 1 (VCAM1). This reduces the trafficking of natural killer (NK) cells to the tumor. VEGF also inhibits the secretion of chemokines such as CXC-chemokine ligands 10 and 11, which would normally attract T cells to the endothelium [42]. Additionally, the altered expression of adhesion molecules facilitates the infiltration of immunosuppressive cells (such as Tregs) into tumoral tissue [44].

Further indirect effects of VEGF in the endothelium include the production FAS antigen ligand (FASL) by the tumor-related blood vessels. This ligand acts as a barrier to CD8 + T-cell infiltration by causing apoptosis of these cells, but without affecting Treg infiltration [42]. Induction of FASL thereby strengthens the tumor’s immune evasion capabilities.

The relationship between the immune system and angiogenesis is bidirectional, so immune cells may also have an impact on angiogenesis. For example, TAMs produce factors that promote lymph-angiogenesis and angiogenesis (in response to hypoxia) [45]. These macrophages release pro-matrix metalloproteinase-9 (pro-MMP-9) a key angiogenesis promoter within the TME [46]. M2 macrophages also release proangiogenic growth factors, such as IL-8 and VEGF [27]. In OC, a high ratio of M2/M1 TAMs is associated with more advanced stages of disease and poor prognosis [47].

**Fig. 3:**
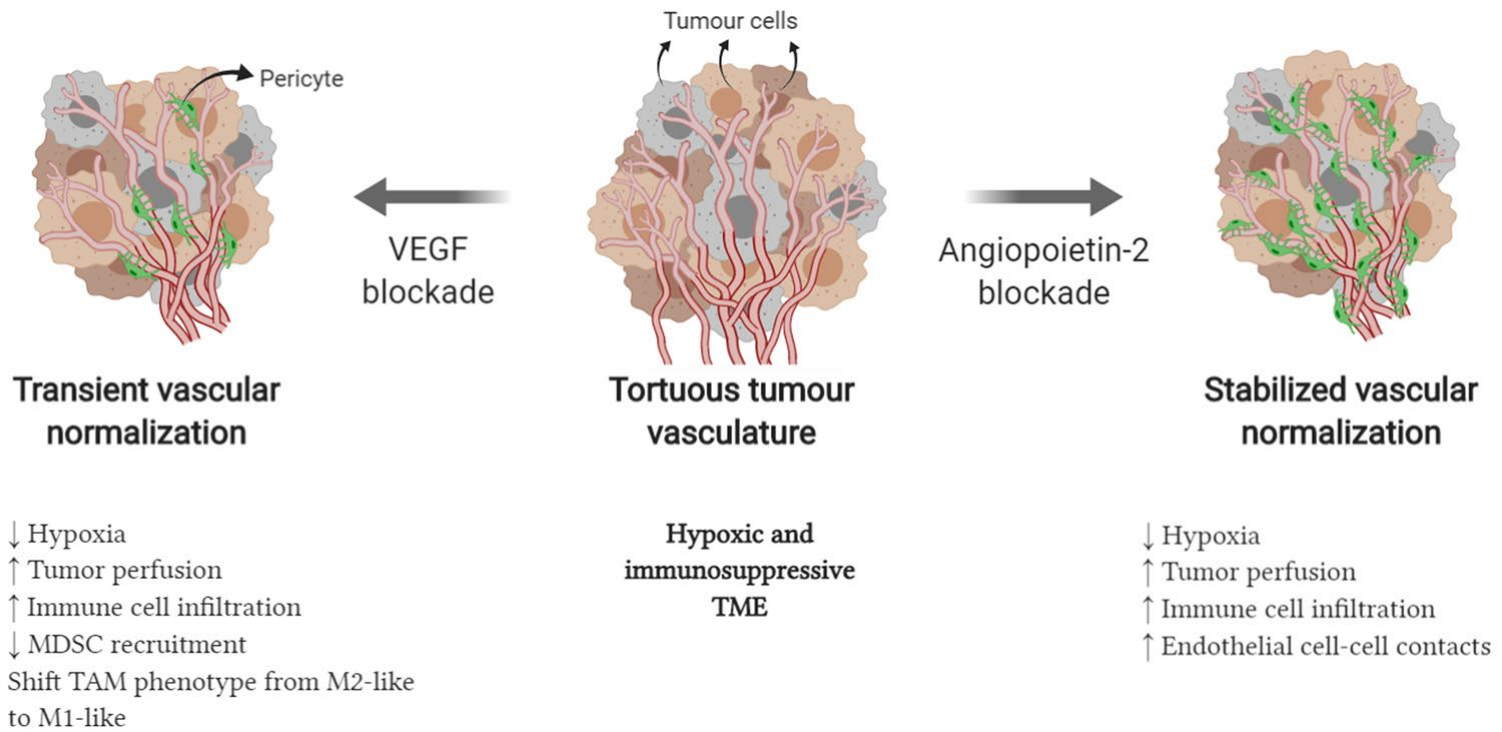
Vascular normalization with antiangiogenic therapy [42]. Modified from Khan and Kerbel, 2018

Antiangiogenic therapy can play a major role in reversing the negative effects of VEGF both in angiogenesis and immune suppression. Experimental research has shown that the administration of bevacizumab can reverse the maturation defect of DCs [48]. In OC cell lines, the inhibition of VEGF production leads to a reduction in the expression of the immunosuppressive ganglioside GD3 and the activation of NK T cells [49]. In addition, blocking VEGF can transiently normalize the tortuous vasculature of the tumor, reducing hypoxia and allowing greater infiltration of immune cells (Fig. 3) [42, 50].

Preclinical data strongly suggest that antiangiogenic treatment facilitates the arrival of immune effectors and reduces the presence of myeloid cells involved in immune suppression, which could translate into a possible synergistic effect with immunomodulators [51]. This has been demonstrated in a number of in vivo cancer models, including breast cancer, pancreatic neuroendocrine tumors, colon cancer, small-cell lung cancer, renal cell carcinoma, and melanoma whereby the administration of immunotherapy (cancer vaccine, adoptive cell therapy, a PD-1 or PD-L1 inhibitor, or a CTLA-4 inhibitor) and antiangiogenic agents showed more marked antitumor activity than the administration of either strategy alone [52, 53, 54, 55, 56, 57, 58, 59]. However, there are few in vivo studies in animal models of OC, probably because the development of genetically engineered animal models of OC has lagged far behind their development in other tumor types [60]. One of the few available animal studies used a murine model of epithelial OC to show that 3TSR (a thrombospondin-1 peptide) induced vascular normalization, which enhanced the effect of an oncolytic virus (Newcastle disease virus) by augmenting immunosuppression within the TME [61]. Animals treated with the combination showed more tumor regression, less ascites development, and a lower rate of metastasis [61].

Soto-Ortiz and Finlay developed an interesting mathematical model with the aim of identifying the therapeutic window of two different approaches to cancer treatment: antiangiogenesis and immunotherapy. The model predicted a synergistic effect of both strategies, the most effective treatment would consist of the combination of an anti-VEGF to interrupt the angiogenic process, limiting tumor growth, and the administration of DCs that would increase the cytotoxicity of CD8 + T cells and would enhance tumor regression [62].

Taken together, these data suggest that antiangiogenic therapy can help shift the OC phenotype from an immunologically “cold” tumor to a “hot” one that is more susceptible to immunotherapy. Clinical studies provide consistent evidence of durable responses with the combination of antiangiogenic therapy and immune checkpoint inhibitors in patients with in other tumor types, such as renal cell carcinoma (RCC) [63], non-small-cell lung cancer [64], or endometrial cancer [65]. To sum up, the theoretical synergism and proven clinical effects in other cancers support clinical investigation of immunotherapy and antiangiogenic therapy in patients with OC.

## Clinical evidence for antiangiogenic therapy in ovarian cancer

There are three groups of antiangiogenic drugs based on their mechanism of action: VEGF inhibitors (bevacizumab), VEGF receptor tyrosine kinase inhibitors (cediranib, pazopanib, sorafenib, and nintedanib), and angiopoietin inhibitors (trebananib) [17]. Data from randomized comparative studies with these agents show a benefit with antiangiogenesis therapy in patients with OC (Tables 1 and 2), but to date, only bevacizumab has been approved in this disease [66, 67, 68, 69, 70, 71, 72, 73,74]. Its approval for use as first-line therapy in combination with chemotherapy and as subsequent maintenance was based on the results of the ICON7 and GOG218 phase III studies [68, 72]. These studies showed that the addition of bevacizumab significantly prolonged progression-free survival (PFS) compared with chemotherapy alone [68, 72]. Although the addition of bevacizumab had no impact on overall survival (OS) in the overall patient populations, OS was increased in the high-risk subgroup of the ICON7 study (defined as patients on stage IV or III with a residual disease after surgery > 1 cm) and in the stage IV subgroup of the GOG218 study [68, 72].

**Table 1:** Randomized comparative studies investigating bevacizumab in ovarian cancer

Study	Phase	Indication	Treatment	*N*	Median PFS, months	PFS HR (95% CI)	Median OS, months	OS HR (95% CI)	ORR (%)	*P* value for ORR
ICON7 [71, 72]	III	First line	Bevacizumab + CT	764	19.9	0.93 (0.83–1.05) *P* = 0.25	58.0	0.99 (0.85–1.14) *P* = 0.85	67	< 0.001
			CT	764	17.5		58.6		48	
GOG0218 [68]	III	First line	Bevacizumab + CT	623	14.1	0.72 (0.63–0.82) *P* < 0.001	39.7	0.92 (0.73–1.15) *P* = 0.45	NA	NA
			CT	625	10.3		39.3			
OCEANS [66, 67]	III	Recurrent, Plat-sens	Bevacizumab + CT	242	12.4	0.48 (0.39–0.61) *P* < 0.0001	33.6	0.95 (0.77–1.18) *P* = 0.65	78.5	< 0.0001
			CT	242	8.4		32.9		57.4	
GOG0213 [69, 70]	III	Recurrent, Plat-sens	Bevacizumab + CT	337	13.8	0.63 (0.53–0.74) *P* < 0.0001	42.2	0.83 (0.68–1.01) *P* = 0.056	78	< 0.0001
			CT	337	10.4		37.3		59	
MITO-168 [73]	III	Recurrent, Plat sens^a^	Bevacizumab + CT	405	11.8	0.51 (0.41–0.64) *P* < 0.001	26.7	1.00 (0.73–1.39) *P* = 0.98	NA	NA
			CT		8.8		27.1		NA	
AURELIA [74]	III	Recurrent, Plat resist	Bevacizumab + CT	179	6.7	0.48 (0.38–0.60) *P* < 0.001	16.6	0.85 (0.66–1.08) *P* < 0.174	30.9	< 0.001
			CT	182	3.4		13.3		12.6	

^a^After first-line bevacizumab. *CI* confidence intervals; *CT* chemotherapy; *HR* hazard ratio; *NA* not available; *NR* not reached; *PEG*-*Lipo* pegylated liposomal; *OS* overall survival; *PFS* progression-free survival; *Plat* platinum; *Resist* resistant; *Sens* sensitive

**Table 2:** Randomized comparative phase III studies investigating antiangiogenic therapies other than bevacizumab in ovarian cancer

Agent (study)	Clinical situation	PFS HR (95% CI)	OS HR (95% CI)
Nintedanib with chemotherapy and as maintenance (AGO-OVAR 12) [75]	First line	0.84 (0.72–0.98) *P* = 0.024	NA
Pazopanib as maintenance only (AGO-OVAR) [79]	First line	0.77 (0.64–0.91) *P* = 0.002	1.08 (0.87–1.33) *P* = 0.499
Cediranib (ICON 6) [76]	Recurrent, platinum-sensitive	0.56 (0.44–0.72) *P* < 0.0001	0.77 (0.55–1.07) *P* = 0.11
Trebananib (TRINOVA-1)^a^ [77, 78, 108]	Recurrent, platinum-free interval 0–12 months	0.70 (0.61–0.80) *P* < 0.001	0.95 (0.81–1.11) *P* = 0.52

All hazard ratios are vs the control group in each study

*CI* confidence intervals; *HR* hazard ratio; *NA* not available; *OS* overall survival; *PFS* progression-free survival

^a^Platinum resistant or only partially sensitive to platinum

Other phase III trials have confirmed the benefit of bevacizumab (added to chemotherapy and as subsequent maintenance) in patients with "platinum-sensitive" and "platinum-resistant" relapses [66, 69, 74]. Results of a recently published randomized study suggest that adding bevacizumab to chemotherapy after platinum-sensitive relapse in patients pretreated with bevacizumab also significantly increases PFS [73].

As described earlier, other phase III studies with positive results have been published with different antiangiogenic agents (Table 2) [75, 76, 77, 78, 79]. However, none of these agents has been submitted to regulatory agencies for approval in OC, suggesting that these agents currently have no clear or obvious advantage over bevacizumab.

## Clinical evidence for immunotherapy in ovarian cancer

Immunotherapy with PD-1 or PD-L1 inhibitors is at an earlier stage in OC compared with other neoplasms, where these agents are now standard treatment, such as melanoma or lung cancer [80, 81]. Therefore, clinical experience with immunotherapies in OC is limited compared with antiangiogenic therapy. To date, all published studies with immunotherapy are phase I or II studies using PD-1 or PD-L1 inhibitors in patients with recurrent OC (Table 3) [82, 83, 84, 85, 86, 87]. The effects of immunomodulators as monotherapy in OC recurrence are modest, with ORRs ranging from 8 to 22% [82, 83, 84, 85, 86, 87]. Although in one study ORR was higher in patients with higher tumor PD-L1 expression [87], the predictive value of PD-L1 in OC is not yet well established.

**Table 3:** Published studies investigating immunotherapies in ovarian cancer

Study	Phase	Indication	Treatment	N	ORR (%)			Median PFS, months	Median OS, months
					All patients	PD-1+ or PD-L1+ patients	PD-1– or PD-L1– patients		
*Pembrolizumab*									
ECHO-202 [85]	I/II	Advanced/ recurrent OC; no prior CI	Pembrolizumab + epacadostat	29	8	NA	NA	NA	NA
KEYNOTE-100 [87]	II	Recurrent platinum-resistant OC	Pembrolizumab	376	8	17.1^**a**^	NA	2.1^b^	17.6^b^
KEYNOTE-028 [86]	Ib	Recurrent treatment-resistant PD-1+ OC, fallopian tube or peritoneal cancer	Pembrolizumab	26	11.5	11.5	–	1.9	13.8
*Atezolizumab*									
Infante et al. [84]	Ia	Recurrent OC	Atezolizumab	12	22	NA	NA	2.9	11.3
*Avelumab*									
JAVELIN [88]	Ib	Recurrent or refractory OC (77% PD-L1+)	Avelumab	124	9.7	12.3	5.9	2.6	10.8
*Nivolumab*									
Hamanishi et al. [83]	II	Platinum-resistant recurrent OC	Nivolumab 1 or 3 mg/kg	20	15	12.5 (n=2/16)	25 (n=1/4)	3.5	20.0

*NA* not available; *OC* ovarian cancer; *ORR* objective response rate; *OS* overall survival; *PD-(L)1* + tumors expressing programmed death protein (ligand)-1; *PD-(L)1–* tumors not expressing programmed death protein (ligand)-1; *PFS* progression-free survival

^a^This is the ORR for the subgroup of patients with a combined positive score (CPS) of ≥ 10, i.e., the highest level of PDL-1 expression (*n* = 82)

^b^PFS and OS data were reported separately for the two study cohorts: cohort A had received 1–3 prior lines of therapy, and were platinum- or treatment-free for 3–12 months at baseline, and cohort B had received 4–6 prior therapy lines and had a platinum- or treatment-free interval at baseline of ≥ 3 months. Median PFS was 2.1 months in both cohorts. Median OS was not reached for cohort A; the median OS reported here is for cohort B

Where reported, PFS in the early studies with PD-1 or PD-L1 inhibitors ranged from 1.9 to 3.5 months, and OS from 10.3 to 20.0 months [82, 83, 84, 86, 88], as would be expected in a heavily pretreated population with advanced disease. Consistent with findings in other tumor types, some patients with OC experience prolonged responses to PD-1 or PD-L1 inhibitors [82, 83, 84, 86].

OC is not a particularly immunogenic cancer, so different approaches have been investigated to convert this tumor into a "hot tumor"—one that is a better target for immune checkpoint inhibitors. One approach that has been trialed is to use chemotherapeutic agents. Adding a PD-L1 inhibitor, avelumab, to treatment with pegylated liposomal doxorubicin (PLD) was no more effective than PLD or avelumab alone in the JAVELIN Ovarian 200 trial in women with platinum-resistant relapsed OC [89].The JAVELIN Ovarian 100 study, which was investigating carboplatin + paclitaxel + avelumab in previously untreated patients with epithelial OC, was stopped prematurely by the sponsor because an interim analysis showed that results did not support the primary hypothesis.

Based on the results obtained so far with PD-1/PD-L1 checkpoint inhibitors in OC, there is a need to test new combinations or sequential therapy to improve these results. Poly ADP ribose polymerase (PARP) inhibitors (olaparib, niraparib, and rucaparib) have been approved by regulatory agencies as maintenance treatment after a response to a platinum combination in recurrent disease, both in patients with and without *BRCA* mutation, after having demonstrated a significant prolongation of PFS. There is emerging preclinical [90] and clinical [91, 92, 93, 94] evidence that the combination of a PARP inhibitor with an antiangiogenic agent or immune checkpoint inhibitor achieves a synergistic effect.

The combination of a PARP inhibitor with a PD-1/PD-L1 inhibitor has been investigated in the MEDIOLA study [95]. This study found that the combination of olaparib with a PD-L1 inhibitor, durvalumab, was generally well tolerated in patients with platinum-sensitive relapsed OC and associated with a 63% ORR [95], which is higher than with other combinations in the same population. Similarly, the TOPACIO/Keynote 162 study reported promising tumor response rates with the combination of the PARP inhibitor niraparib and the PD-1 inhibitor pembrolizumab in patients with platinum-resistant relapsed OC [96]. Ongoing phase III studies with combinations of a PARP inhibitor and immunotherapy will confirm whether this approach is superior to standard treatment options.

## Clinical evidence for combination of antiangiogenic therapy and immunotherapy

Because of the biological synergism between angiogenesis and tumor-related immune responses, the combination of antiangiogenic therapy and immunotherapy has been investigated in a range of solid tumors, including melanoma [97], RCC [98], biliary tract cancer [99], non-small-cell lung cancer [100, 101], and glioblastoma [102]. Although remarkable responses have been reported in individual cases [103], ORRs in clinical trials have been variable, ranging from 4% with the combination of ramucirumab and pembrolizumab in patients with biliary tract cancer [99] to 55% with the combination of sunitinib and nivolumab in patients with RCC [98]. In addition, the toxicity profile differs markedly between combinations. Ipilimumab + bevacizumab appears to have a manageable toxicity profile [97, 102]. However, nivolumab + sunitinib was poorly tolerated, with a high incidence of Grade 3 or 4 adverse events (~ 70 to 80%) [98, 101].

To date, data on the combination of antiangiogenic therapy and immunotherapy in patients with OC are limited. A case report described a marked and long-lasting response in a patient with relapsed OC who received nivolumab and pazopanib. Interesting, one of the prior regimens this patient had received (and responded to) was a PARP inhibitor (olaparib) with nivolumab [104]. A phase I dose-escalation study was published investigating two combinations, durvalumab + cediranib and durvalumab + olaparib in patients with gynecologic cancers, including 19 with OC [105]. Six of the 12 patients treated with durvalumab + cediranib had a partial response, and these partial responses lasted for at least 5 months. Similar antitumor activity was seen in a recent study of nivolumab + bevacizumab in patients with recurrent OC (20 who were platinum-sensitive and 18 platinum-resistant). Overall, eight patients had a confirmed partial response, and another six had stable disease lasting ≥ 6 months. Median PFS was 9.4 months [106]. Recently, the results of a phase II study with the combination of bevacizumab + pembrolizumab and metronomic cyclophosphamide in 40 women with recurrent OC were presented. The patients were heavily treated, having received a median of five previous lines of treatment. This combination was associated with an ORR of 40% and a disease control rate of 95%. In this study, the 6-month PFS rate was 100% in patients who had platinum-sensitive disease and 59% in those who did not [107].

Since bevacizumab is approved for use in OC, and appears well tolerated when used in combination with immunotherapy in other indications, a number of phase II and phase III clinical studies are currently underway to investigate the use of bevacizumab in combination with immunotherapeutic agents (Table 4).

**Table 4:** Ongoing studies with bevacizumab and immunotherapies in patients with ovarian cancer ( Source: https://clinicaltrials.gov)

Study ID (study name)	Phase	Indication	Combination regimen being investigated	Comparator arm(s)	*N*	Primary endpoint	Expected completion date
*First-line therapy*							
NCT03038100 (IMagyn050)	III	Newly diagnosed Stage III-IV OC, primary peritoneal cancer and/or fallopian tube cancer	Bevacizumab + atezolizumab + CT	Bevacizumab + CT	1300	PFS and OS	December 2021
*Relapsed, platinum-sensitive ovarian cancer*							
NCT02891824 (ENGOT Ov29/ATALANTE)	III	Platinum-sensitive recurrent epithelial OC, primary peritoneal cancer and/or fallopian tube cancer with platinum-free interval > 6 months)	Bevacizumab + atezolizumab + CT	Bevacizumab + CT	405	PFS	September 2023
NCT03596281 (PEMBOV)	I	Recurrent platinum-sensitive OC, primary peritoneal cancer and/or fallopian tube cancer	Bevacizumab + pembrolizumab	Pembrolizumab + PEG-liposomal doxorubicin	40	DLT	June 2024
*Relapsed platinum-resistant or refractory ovarian cancer*							
NCT03353831 (AGO-OVAR 2.29)	III	Recurrent OC, fallopian tube, or primary peritoneal cancer with 1st or 2nd relapse < 6 months after platinum-based chemotherapy or 3rd relapse	Atezolizumab + bevacizumab + CT	Bevacizumab + CT	664	OS and PFS	September 2022
NCT03574779	II	Recurrent PARP inhibitor-naïve, platinum-resistant OC, primary peritoneal cancer and/or fallopian tube cancer	Bevacizumab + TSR-042 (PD-1 inhibitor) + niraparib	–	40	ORR	June 2020
NCT03363867 (BEACON)	II	C1 subtype of platinum-resistant or refractory recurrent OC, fallopian tube or peritoneal cancer	Bevacizumab + atezolizumab + cobimetinib	–	29	ORR	July 2020
NCT02659384	II	Recurrent platinum-resistant advanced or metastatic OC, fallopian tube or peritoneal cancer	Atezolizumab + bevacizumab ± aspirin	Bevacizumab Atezolizumab ± aspirin	160	PFS	January 2021
NCT02923739	II	Platinum-resistant recurrent epithelial OC, primary peritoneal cancer and/or fallopian tube cancer	Bevacizumab + emactuzumab + paclitaxel	Bevacizumab + paclitaxel	121	Safety and PFS	May 2025
*Relapsed ovarian cancer, either platinum sensitive or platinum resistant*							
NCT02853318 (results available [107])	II	Recurrent OC, fallopian tube or primary peritoneal cancer	Bevacizumab + pembrolizumab + cyclophosphamide	–	40	AEs and PFS	March 2019
NCT02873962	II	Recurrent OC, fallopian tube or peritoneal cancer	Bevacizumab + nivolumab	–	38	ORR	February 2024
*Relapsed ovarian cancer, platinum-sensitivity status unclear*							
NCT03197584 (QUILT-3.051)	Ib/II	Recurrent epithelial OC	Bevacizumab + avelumab + CT + cancer vaccines	–	67	AEs and ORR	April 2019

*AE* adverse event; *CT* chemotherapy; *DLT* dose-limiting toxicity; *ID* identifier; *OC* ovarian cancer; *ORR* objective response rate; *OS* overall survival; *PEG* pegylated; *PFS*: progression-free survival

In addition to these, several ongoing Phase III first-line trials are currently evaluating the possible benefit of adding checkpoint inhibitors to chemotherapy, followed by maintenance therapy with these plus a PARP inhibitor. However, only one of these trials requires mandatory use of bevacizumab (DUO-O/ENGOT Ov46 study) (Table 5). Although the use of bevacizumab is optional and at the investigator's discretion in two other studies (First/ENGOT Ov44 and ENGOT Ov43), it is expected that their protocols will be revised to make treatment with bevacizumab mandatory, in light of the results of the Javelin 100 and Javelin 200 studies. The fourth study (Athena/ENGOT Ov45) explores an immunotherapy and/or a PARP inhibitor only in a maintenance setting without bevacizumab. The aim of these studies is to assess the potential synergism between PARP inhibitors and immunotherapy (plus bevacizumab in some of them), since adding only immunomodulators to chemotherapy has not shown benefit in OC.

**Table 5:** Ongoing front-line studies with immunotherapies and PARP inhibitors ± bevacizumab in advanced ovarian cancer ( Source: https://clinicaltrials.gov)

Study ID (study name(s))	Treatment purpose	Bevacizumab	Target N	Treatment	Maintenance (where applicable)
NCT03737643 (DUO-O/ENGOT Ov46)	Front line and maintenance	Mandatory, but optional in patients with *BRCA* mutation	1056	CP + bevacizumab CP + bevacizumab + durvalumab CP + bevacizumab + durvalumab CP + bevacizumab + durvalumab	Bevacizumab Bevacizumab + durvalumab Bevacizumab + durvalumab + olaparib Bevacizumab + durvalumab + olaparib
NCT03740165 (KEYLYNK-001/ENGOT Ov43)	Front line and maintenance	Optional^a^	1086	CP + pembrolizumab CP + pembrolizumab CP + PL-pembrolizumab	Pembrolizumab + olaparib Pembrolizumab + PL-olaparib PL-pembrolizumab + PL-olaparib
NCT03602859 (FIRST/ENGOT Ov44)	Front line and maintenance	Optional^a^	912	CP + dostarlimab CP + PL-dostarlimab CP + PL-dostarlimab	Dostarlimab + niraparib PL-dostarlimab + niraparib PL-dostarlimab + PL-niraparib
NCT03522246 (ATHENA/GOG 3020/ENGOT Ov45)	Only maintenance after front line^c^	Prohibited during maintenance	1012		Rucaparib + nivolumab Rucaparib + PL-nivolumab PL-rucaparib + nivolumab PL-rucaparib + PL-nivolumab

*CP* carboplatin + paclitaxel; *ENGOT* The European Network for Gynaecological Oncological Trial groups; *PL* placebo

^a^Likely to become mandatory (see “Discussion” section)

^b^*BRCA*-mutated patients will be randomized only to the active treatment arms, and not to the standard of care (carboplatin + paclitaxel) plus placebo arm

^c^Front-line platinum-based chemotherapy; patients were required to have a response to front-line treatment

As well as defining the efficacy and safety profile of antiangiogenic and immunotherapy combinations, future research needs to validate predictive biomarkers for these combinations in OC and clarify the molecular basis for resistance to immunotherapy.

## Conclusions

There is a strong biological rationale for combining immunotherapy with antiangiogenic agents in the treatment of OC, but clinical experience with this combination is limited so far. The results of ongoing studies will clarify the role of such combinations in patients with OC.
